# Gelidiales Are Not Just Agar—Revealing the Antimicrobial Potential of *Gelidium corneum* for Skin Disorders

**DOI:** 10.3390/antibiotics11040481

**Published:** 2022-04-05

**Authors:** Margarida Matias, Susete Pinteus, Alice Martins, Joana Silva, Celso Alves, Teresa Mouga, Helena Gaspar, Rui Pedrosa

**Affiliations:** 1 MARE—Marine and Environmental Sciences Centre, Polytechnic of Leiria, 2520-630 Peniche, Portugal; maggiemmatias@gmail.com (M.M.); alice.martins@ipleiria.pt (A.M.); joana.m.silva@ipleiria.pt (J.S.); celso.alves@ipleiria.pt (C.A.); mougat@ipleiria.pt (T.M.); 2 BioISI—Biosystems and Integrative Sciences Institute, Faculty of Sciences, University of Lisbon, 1749-016 Lisboa, Portugal; hmgaspar@fc.ul.pt; 3 MARE—Marine and Environmental Sciences Centre, ESTM, Polytechnic of Leiria, 2520-630 Peniche, Portugal

**Keywords:** marine natural products, seaweeds, skin microbiota, dermatological applications, antimicrobial activity, Rhodophyta, acne vulgaris, skincare, *Staphylococcus epidermidis*, red algae

## Abstract

In recent decades, seaweeds have proven to be an excellent source of bioactive molecules. Presently, the seaweed *Gelidium corneum* is harvested in a small area of the Portuguese coast exclusively for agar extraction. The aim of this work was to fully disclosure *Gelidium corneum* as a sustainable source of antimicrobial ingredients for new dermatological formulations, highlighting its potential to be explored in a circular economy context. For this purpose, after a green sequential extraction, these seaweed fractions (F1–F5) were chemically characterized (^1^H NMR) and evaluated for their antimicrobial potential against *Staphylococcus aureus*, *Staphylococcus epidermidis* and *Cutibacterium acnes*. The most active fractions were also evaluated for their effects on membrane potential, membrane integrity and DNA damage. Fractions F2 and F3 displayed the best results, with IC_50_ values of 16.1 (7.27–23.02) μg/mL and 51.04 (43.36–59.74) μg/mL against *C. acnes*, respectively, and 53.29 (48.75–57.91) μg/mL and 102.80 (87.15–122.30) μg/mL against *S. epidermidis*, respectively. The antimicrobial effects of both fractions seem to be related to membrane hyperpolarization and DNA damage. This dual mechanism of action may provide therapeutic advantages for the treatment of skin dysbiosis-related diseases.

## 1. Introduction

In recent decades, marine organisms have proven to be an excellent source of bioactive molecules with a wide range of applications. However, few examples have reached the industrial sector, particularly due to the limitations of biomass availability, solvent suitability, low yields of extraction, and specific legal requirements, among others. The seaweed *Gelidium corneum* (former *Gelidium sesquipedale*) is a Rhodophyta belonging to the order Gelidiales, and several species belonging to this order, including *Gelidium corneum*, have the characteristic of being rich in agarans, highly valued in the food industry [1,2]. Presently, this seaweed is harvested in a small area of the Portuguese coast São Martinho do Porto for agar extraction. However, *Gelidium* species are rich in bioactive compounds such as mycosporine-like amino acids, flavonoids, pigments, phycobiliproteins, fatty acids, etc., with relevant biotechnological potential [3,4,5,6]. Within a circular economy approach it is highly relevant to understand the full potential of this biomass, to take the maximum advantage of one resource for the development of multiple new products, enhancing economic revenues and, consequently, boosting the local economy.

Within this framework, the present study targeted the red seaweed *Gelidium corneum* as a source of bioactive ingredients with antimicrobial activity with relevance for inclusion in new dermatological formulations.

Skin is the most effective barrier of the human body against external aggressions. Covered by a highly complex microbiome composed of bacteria, fungi and viruses, this living barrier is an effective defence against invading pathogens and heavily contributes to modulating the immune system [7]. Some of the most representative species of skin microbes include *Cutibacterium acnes* (former *Propionibacterium acnes*), *Staphylococcus* spp., *Streptococcus* spp., *Corynebacterium* spp., and *Malassezia* spp., which are distributed according to their specific affinity for a determined microenvironment—moist, dry or sebaceous [7]. While in normal conditions these microorganisms are fundamental for a healthy skin barrier, changes in their normal balance (dysbiosis) may lead to the development of skin pathologies such as acne vulgaris, dermatitis, eczema, and chronic wounds, among others [7]. Even though the skin’s microbiome is composed of thousands of different microorganisms, recent studies have focussed on the relationships between two commensal bacteria, *Staphylococcus epidermidis* and *Cutibacterium acnes* [8,9]. The dysbiosis between these two organisms results in a series of complications for the human host. Oily skin or excessive sebum production by the oil glands, may boost the growth of *C. acnes* that, combined with a decreased *S. epidermidis* population, may lead to a skin pathological condition known as acne vulgaris [10,11]. Acne vulgaris is a chronic inflammation of the skin, beginning in the pilosebaceous unit. Not only it is triggered by the increase in sebum production, but also by the hypercornification of the pilosebaceous glands due to the hyperproliferation of keratinocytes on the upper part of the follicle.

The homeostasis of the skin microbiota is considered to be the objective of any acne treatment [10,12]. As a consequence of microbial imbalance, the treatments available are mostly provided by topical antibiotic prescription; however, severe cases require oral treatment. The most common antibiotics for acne topical treatment are tetracycline, clindamycin, and erythromycin, sometimes combined with benzoyl peroxide and zinc acetate [13,14,15]. A prescription of oral antibiotics is mostly avoided due to the possibility of increased antibiotic resistance. However, *C. acnes* infections can also occur in other organs due to wounds and, in these cases, there is not any other treatment option.

When the balance of these two commensals is disrupted there can also occur an excess of *S. epidermidis* growth, which may result in nosocomial infections [16]. Although *S. epidermidis* rarely lead to severe life-threatening diseases, their infections are extremely common and difficult to treat. This microorganism is frequently involved in vascular graft, prosthetic joint, and cardiac device infections, among others, being the second infectious driver after *Staphylococcus aureus* [16].

The skin microbiome has a vast influence on individuals’ wellbeing, thus, finding natural derivatives that can not only deter pathogens but also maintain a microbial balance by targeting key microbes is critical [7].

Knowing that marine seaweeds are a propelling source of new chemical structures with an array of bioactivities, including antimicrobial activity [17,18], this work aims to disclose the antimicrobial potential of *G. corneum*-derived components against microorganisms that are frequently associated with skin disorders, namely *Staphylococcus aureus*, *Staphylococcus epidermidis* and *Cutibacterium acnes*.

## 2. Results

### 2.1. Chemical Screening by ^1^H NMR

**Figure 1:**
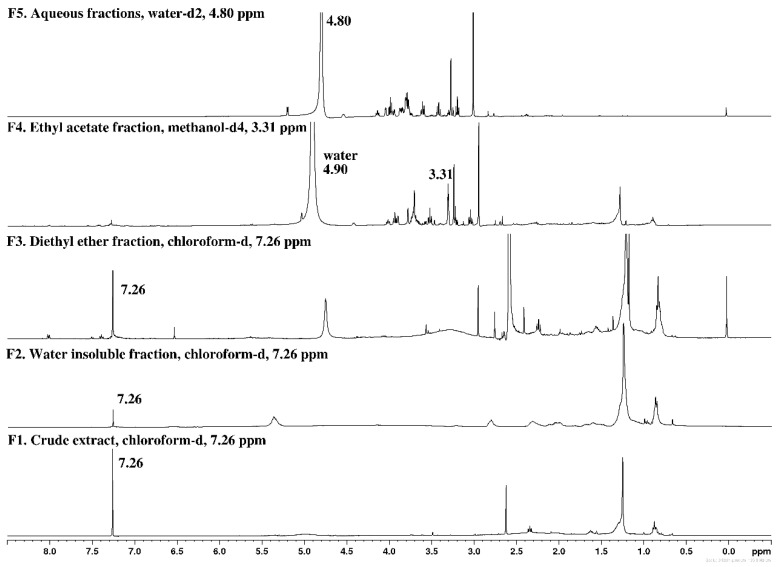
^1^H NMR (400 MHz) spectra of *Gelidium corneum* fractions (F1–F5).

The chemical profile of the fractions obtained from *Gelidium corneum* was evaluated by ^1^H NMR and the corresponding spectra are depicted in Figure 1.

Crude extract (F1) evidenced signals in the region of 0.8–2.8 ppm, characteristic of less polar lipophilic compounds such as fatty acids, sterols, terpenes, pigments, and other lipids [19,20]. The presence of these classes of compounds was also evidenced in the insoluble fraction retained in the filter (F2), with the intensity of signals being more pronounced in this fraction due to their higher concentration. More intense proton signals in the region of 0.8–3.1 ppm were also observed in diethyl ether fraction (F3), also suggesting the richness of lipophilic compounds in this fraction. Fraction F3 also evidenced low-intensity signals of aromatic protons (7.5–8.2 ppm), which can be attributed to phenolic compounds. The signals of these compounds are also visible in the spectrum of ethyl acetate fraction (F4), although with less intensity. In this fraction, the most intense signals were observed at 2.7–3.9 ppm, which can be attributed to the presence of amino compounds such as amino acids and proteins [20,21]. However, signals between 3.2 and 4.1 ppm were observed in fractions F4 and F5, which can denote the presence of alcohols, sugars, and esters [20]. Signals between 3.0 and 4.4 ppm are characteristic of the ring hydrogens of polysaccharides [22], supporting the richness of the aqueous fraction (F5) in this group of molecules.

### 2.2. Antimicrobial Activity of Gelidium corneum Fractions

**Figure 2:**
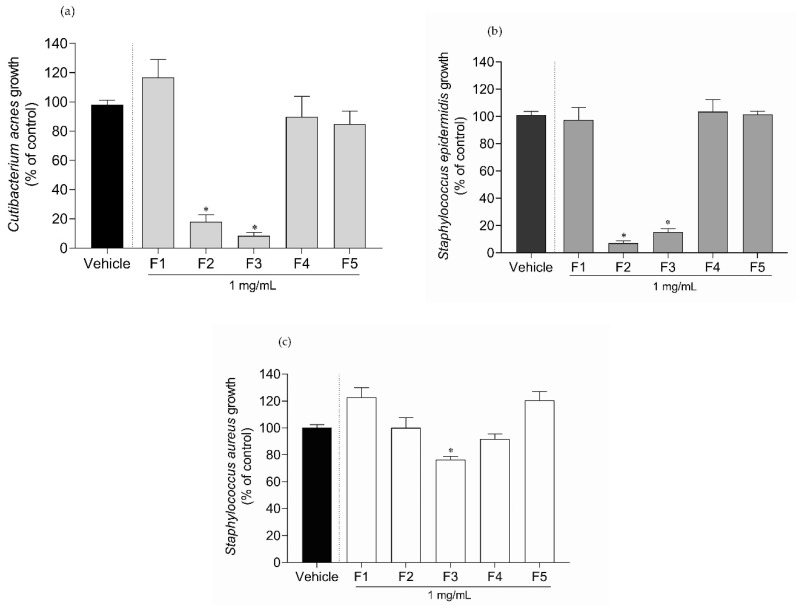
Antimicrobial activity of *Gelidium corneum* fractions (1000 μg/mL) against (**a**) *Cutibacterium acnes*, (**b**) *Staphylococcus aureus*, and (**c**) *Staphylococcus epidermidis*. Values in each column represent the mean ± SEM of three independent experiments carried out in triplicate. Symbol (*) represents significant differences when compared to vehicle (ANOVA, Dunnett’s test, *p* < 0.05).

The antimicrobial potential of *G. corneum* fractions was tested against *C. acnes*, *S. epidermidis* and *S. aureus* growth. The results are shown in Figure 2.

Only the water-insoluble fraction (F2) and diethyl ether fraction (F3) inhibited *C. acnes* and *S. epidermidis* growth by more than 50%. In the case of *S. aureus*, only the F3 fraction promoted inhibition (≈25%). Since fractions F2 and F3 showed high inhibitory activity, a dose–response analysis was conducted and the IC_50_ determined. The results are shown in Table 1.

*Gelidium corneum* F2 and F3 fractions exhibited IC_50_ values of 53.29 (48.75–57.91) μg/mL and 102.8 (87.15–122.30) μg/mL, respectively, against *S. epidermidis*. Concerning *C. acnes*, fraction F2 revealed the highest potency with an IC_50_ of 16.10 (7.27–23.02) μg/mL, followed by fraction F3 with an IC_50_ of 51.04 (43.36–59.74) μg/mL.

### 2.3. Effects of Gelidium corneum Fractions on Cutibacterium acnes and Staphylococcus epidermidis Membrane Potential

**Figure 3:**
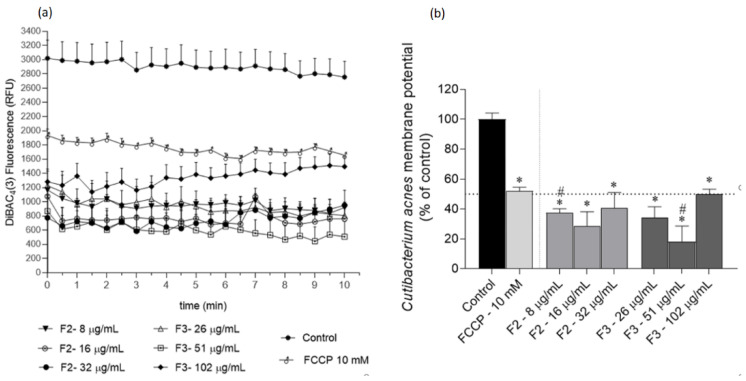
*Cutibacterium acnes* membrane potential when exposed to *Gelidium corneum* F2 (water insoluble) and F3 (diethyl ether) fractions at ½ IC_50_; IC_50_ and 2 × IC_50_, labelled with DiBAC_4_(3) probe; (**a**) 30 s interval readings; (**b**) after 10 min. FCCP (10 mM) was used as positive control and DMSO as negative control. Each value and bars represent the average of three independent experiments. Vertical lines represent the standard error of the mean (SEM). Symbol (*) represents significant differences when compared to the control. Symbol (^#^) represents significant differences when compared to FCCP (ANOVA, Dunnett’s test, *p* < 0.05).

**Figure 4:**
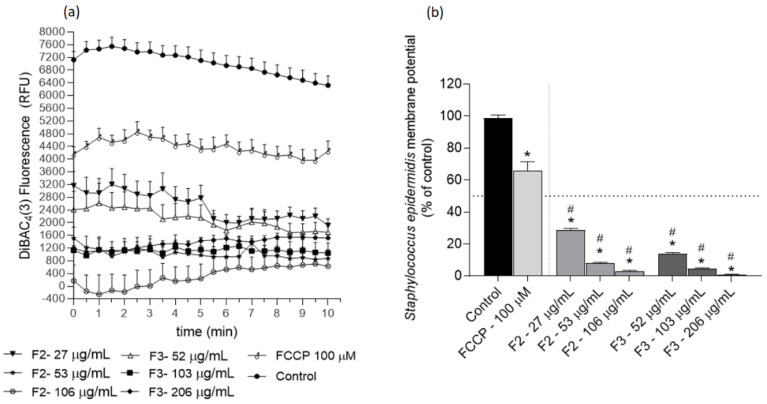
*Staphylococcus epidermidis* membrane potential when exposed to *Gelidium corneum* F2 (water insoluble) and F3 (diethyl ether) fractions at ½ IC_50_; IC_50_ and 2 × IC_50_, labelled with DiBAC_4_(3) probe; (**a**) 30 s interval readings; (**b**) after 10 min. FCCP (100 µM) was used as positive control and DMSO as negative control. Each value and bars represent the average of three independent experiments. Vertical lines represent the standard error of the mean (SEM). Symbol (*) represents significant differences when compared to the control. Symbol (^#^) represents significant differences when compared to FCCP (ANOVA, Dunnett’s test, *p* < 0.05).

Several assays were conducted to understand the possible mechanisms underlying the F2 and F3 fractions’ antimicrobial effects. The effects on *C. acnes* and *S. epidermidis* membrane potential are depicted in Figure 3 and Figure 4, respectively.

The results obtained with the DiBAC_4_(3) method suggest that F2 and F3 fractions promote a membrane hyperpolarization in *C. acnes* and *S. epidermidis*, which is noticeable due to the lower fluorescence emission when compared with the control. Additionally, the fractions exhibited a more marked effect than the positive control FCCP in both bacteria. In *C. acnes*, both fractions decreased the membrane potential in more than 50%, except for F3 at 2 × IC_50_ after 10 min (Figure 3b). This profile was similar against *S. epidermidis*; however, the hyperpolarization was more pronounced, with a reduction in membrane polarization ranging from ≈70% (fraction F2 at ½ IC_50_) to ≈100% reduction (fraction F3 at 2 × IC_50_).

### 2.4. Effects of Gelidium corneum Fractions on Cutibacterium acnes and Staphylococcus epidermidis Membrane Integrity

**Figure 5:**
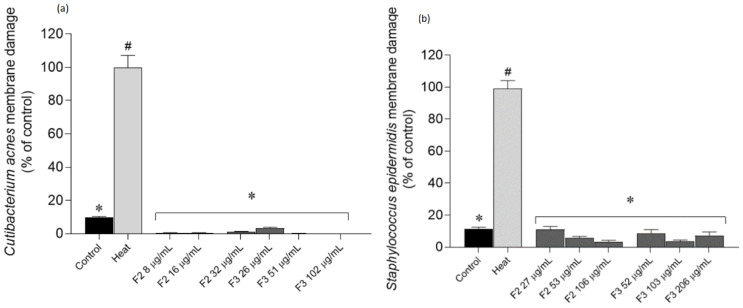
*Cutibacterium acnes (***a**) and *Staphylococcus epidermidis* (**b**) membrane integrity when exposed to *Gelidium corneum* F2 and F3 fractions at ½ IC_50_; IC_50_ and 2 × IC_50_, labelled with Sytox Green probe. Bacterial cells exposed to a heat treatment were used as a positive control. DMSO was used as negative control. Each value represents the average of three independent experiments. Bars represent the standard error of the mean (SEM). Symbols represent significant differences when compared to the heat-treated cells (*) and (^#^) to control (ANOVA, Dunnett’s test, *p* < 0.05).

To further understand if fractions could impair bacterial growth by affecting the membrane integrity, Sytox Green—a green-fluorescent nuclear probe—was used. This probe is impermeant to cells; however, when the membrane is disrupted, this probe links to nucleic acids emitting fluorescence, thus being an extremely useful tool to monitor membrane damage (Figure 5).

After an incubation with the most active fractions, it was possible to verify that they did not promote membrane damage, since there were no significant differences in relation to the untreated control.

### 2.5. Potential of Gelidium corneum Fractions to Promote DNA Damage

**Figure 6:**
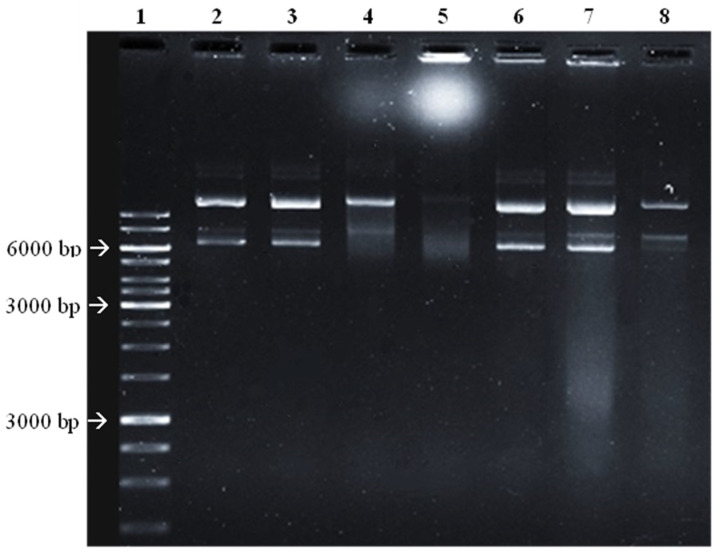
Electrophoresis gel. 1—Marker; 2—DNA; 3—negative control (DMSO); 4—10 µg/mL ciprofloxacin; 5—30 µg/mL ciprofloxacin; 6—negative control (DMSO); 7—F2 (water insoluble) fraction (1 mg/mL); 8—F3 (diethyl ether) fraction (1 mg/mL). Agarose at 0.8%, ran at 85 V for 1 h. Image was obtained through gel imaging system (Gel doc). This figure is representative of three independent experiments.

Since different mechanisms can be behind the antimicrobial effects, the ability of the most active fractions to link and damage DNA was evaluated. The results are shown in Figure 6.

In Figure 6, it is possible to observe a clear smear of the DNA bars in the *G. corneum* F2 and F3 (lanes 7 and 8) when compared to the DMSO controls. Additionally, in lane 8 (F3), both linear and supercoiled DNA have a lower intensity, suggestive of DNA degradation. Ciprofloxacin promoted extensive DNA damage at 30 µg/mL and, at 10 µg/mL, the degradation is particularly evident in the supercoiled DNA (lane 4).

## 3. Discussion

In the present work, *Gelidium corneum* was evaluated regarding its potential as a source of antimicrobial compounds through a green extraction approach, aiming to develop new therapeutic alternatives for dysbiosis-related skin disorders. It was verified that two fractions—F2 (water insoluble fraction) and F3 (diethyl ether fraction)—were particularly active against *S. epidermidis* and *C. acnes*.

One of the main causes behind dermatology appointments is acne vulgaris. This disease is the most common skin problem worldwide, occurring in adulthood in about 50% of the population in occidental countries [23,24,25]. On the other hand, *S. epidermidis* can be an important opportunistic pathogen, with treatment options posing a challenge. Most infections caused by this microorganism start with the introduction of skin bacteria during the insertion of a medical device into the patient [16]. When looking at therapeutical options targeting both microorganisms, the conventionally prescribed topical and oral antibiotics can have some serious side effects, such as disrupting gut health and increasing skin dryness, impacting patients’ quality of life. On the other hand, even when the side effects are minimal, bacterial resistance might still occur [26]. This makes seeking new antibiotic options urgent. There are several natural products that could be possible candidates for novel drugs targeting multiple pathogenic factors [27]. Although in the present work the antimicrobial activity against *S. aureus* was low, previous studies with other *Gelidium* species have shown the capacity to deter the growth of *S. aureus* [28,29,30,31]. Additionally, a review gathered by Pérez, Falqué and Domínguez [31] cited *G. attenatum*, *G. micropterum*, *G. pulchellum*, *G. pusillum*, *G. robustum,* and *G. spinosum* with antimicrobial activity against different bacteria, including *Escherichia coli*, *Klebsiella pneumoniae*, *Vibrio* spp. and *Enterococcus faecalis*. However, no studies were found with *Gelidium* species against *C. acnes* or *S. epidermidis*. Choi et al. [32] reported 15 seaweed species with antimicrobial activity against *C. acnes*, with the red seaweed *Symphyocladia latiuscula* displaying the highest inhibitory potential (MIC = 160 µg/mL). Comparatively, the results obtained with F2 and F3 fractions from *G. corneum* are quite exciting due to their high potency, particularly against *C. acnes*; thus, they are promising candidates for use in new formulations aiming to treat skin diseases, where *C. acnes* and *S. epidermidis* play a key role. Scientific evidence suggests that *S. epidermidis* has a suppressive effect against *C. acnes*, and thus extracts with a higher antimicrobial effect against *C. acnes* than against *S. epidermidis* may bring additional advantages for acne vulgaris therapeutics [33]. This evidence highlights the relevance of the results obtained with *G. corneum* F2 and F3 fractions.

Antibiotics can work in a synergic way to effectively decrease bacterial resistance. Yet, their mechanism and range of action should be complementary. This is already exploited specifically in acne vulgaris treatment with the combination of different oral antibiotics and benzoyl peroxide [15]. To better understand the antibacterial effect, it is necessary to evaluate the possible mechanisms of action which can be underlying the bacterial growth inhibition. Since fractions F2 and F3 revealed promising results, the antimicrobial effects due to membrane potential disruption, membrane rupture, and/or DNA damage were evaluated.

Membrane potential is central to bacterial development; therefore, disruptions to it might induce an antimicrobial effect [34]. Across the cellular membrane, there is an electrochemical potential that is involved in several functions of bacterial cells, such as intra- and intercellular signalling mediation, which in turn regulates important physiological processes, namely mechano-sensation, spore formation, and biofilm dynamics [35,36,37,38]. In addition to its role in bioelectrical signalling, membrane potential is also central to cellular proliferation since it provides the essential driving force for ATP synthesis [39], which is crucial for cell division [40]. Although this potential may fluctuate depending on cells’ physiologies, abrupt changes may lead to cellular death. In particular, several studies have shown that a hyperpolarized membrane is associated with bacterial death [34,41]. It was verified that the most active extracts (F2 and F3) promoted a hyperpolarization of bacteria membrane potential (Figure 3 and Figure 4). Although studies surrounding the mechanisms of action of seaweed derivatives are scarce, Patra et al. [42] evaluated the mechanisms underlying the antimicrobial effects of essential oils extracted from *Ulva linza* (former *Enteromorpha linza)* against *Escherichia coli* and verified that the antimicrobial effects were related to changes in the membrane potential. Concerning phytochemical sources, Wu et al. [43] evaluated the antimicrobial effects of a natural compound (2*R*,3*R*-dihydromyricetin) obtained from the pine needles of *Cedrus deodara* against *S. aureus* and verified that this compound promoted a high membrane hyperpolarization, resulting in bactericidal effects. Although the results shown in the present work suggest that *G. corneum* fractions did not promote membrane damage, other seaweed species have shown antimicrobial activity mediated by membrane rupture. Patra and Baek [44] evaluated the antimicrobial mechanisms of oil extracted from the seaweed *U. linza* against *Bacillus cereus* and *S. aureus* and verified that the antimicrobial effects were related to membrane injury. Additionally, El Shafay et al. [45] evaluated the antimicrobial activity of *Sargassum* species against *S. aureus* and *Klebsiella pneumoniae* and concluded that the antimicrobial activity was mediated by cells’ membranes rupture and physical distortions. A work conducted with the green seaweed *U. linza* showed that essential oil from this seaweed promotes membrane damage in the foodborne pathogen *Escherichia coli* [44].

It has been suggested that antimicrobial compounds can reach DNA through membranes, with or without membrane rupture [46,47]. Thus, although it was not possible to verify membrane damage, a simple test was conducted to understand if the seaweed fractions could impact DNA. It was shown that both fractions F2 and F3 can promote DNA damage. In accordance with these results, Pinteus et al. [48] also showed that extracts derived from the red seaweed *Asparagopsis armata* had potential to promote DNA damage. Additionally, seaweed polysaccharides have shown antimicrobial activity mediated by DNA damage [49]. Other natural products from various works have also shown the ability to target DNA. Subramanian et al. [50] verified that resveratrol inhibited the growth of *E. coli*, probably by inducing DNA damage. Da et al. [51] evaluated the antimicrobial activity of an extract obtained from *Scutellaria baicalensis* root and verified that it had antimicrobial activity against fungi, possible mediated by DNA damage. Antimicrobial peptides extracted from different sources have also shown potential to target bacteria and fungi DNA [52]. He et al. [53] isolated a novel polysaccharide from *Streptomyces virginia* and verified that this compound exhibited antimicrobial activity against several microorganisms, possibly mediated by DNA damage.

Fractions 2 and 3 from *Gelidium corneum* are rich in lipophilic compounds, as evidenced by their ^1^H NMR spectra. Lipophilic compounds from different algae species have shown to display antimicrobial effects [31,49,54,55,56] and within this group of compounds, different terpenes sourced from red algae show great potential. Rodrigues et al. [57] studied the antimicrobial activity of terpenes isolated from *Sphaerococcus coronopifolius* and one sphaerane bromoditerpene had a great effect on *S. aureus* with an IC_50_ of 6.35 µM. Xu et al. [55] reported that tetracyclic brominated diterpenes from the same algae exhibited a bactericidal effect with MIC values of 16 and 128 μg/mL against multi-resistant *Staphylococcus aureus*. Amongst lipophilic compounds, fatty acids from different species of seaweeds have a reported antimicrobial effect in several microorganisms. For example, *Dunaliella salina* fatty acids are described for their effect over *Listeria monocytogenes* and *B. cereus* with a MIC value of 2.5 mg/mL, and over *Salmonella enteritidis* with a MIC of 1.25 mg/mL [22]. There are no studies regarding compounds isolated from red algae affecting the growth of *S. epidermidis* and *C. acnes*, highlighting the importance of *G. corneum* as a source of compounds with antimicrobial effects against these bacteria. The chemical characterization here presented constitutes the first approach concerning the evaluation of *G. corneum* ingredients for dermatological applications, also validating the effectiveness of the fractionation methodology here reported. Yet, it is important to proceed with a deeper chemical characterization to identify the compound(s) responsible for the observed antimicrobial activities acting individually and/or synergistically.

## 4. Materials and Methods

### 4.1. Seaweed Harvest and Sampling

*Gelidium corneum* (Hudson) J.V.Lamouroux 1813 was collected on October 2020 at Praia dos Barcos (39°22′35.9″ N 9°20′23.7″ W), Peniche, Portugal (identification number: GC.PB.2020-10). It was identified by Professor Teresa Mouga, a botanical expert, and immediately transported to the laboratory facilities (MARE-Polytechnic of Leiria). Samples were washed, firstly with sea water and then with distilled water, to remove invertebrate organisms and debris, and dried at 70 °C with air circulation (Universal laboratory oven UF450, Memmert, Buchenbach, Germany). Dried samples were ground (Moulinex Food processor, Paris, France) and stored in a dry dark place until extraction processing.

### 4.2. Extraction Procedure

**Figure 7:**
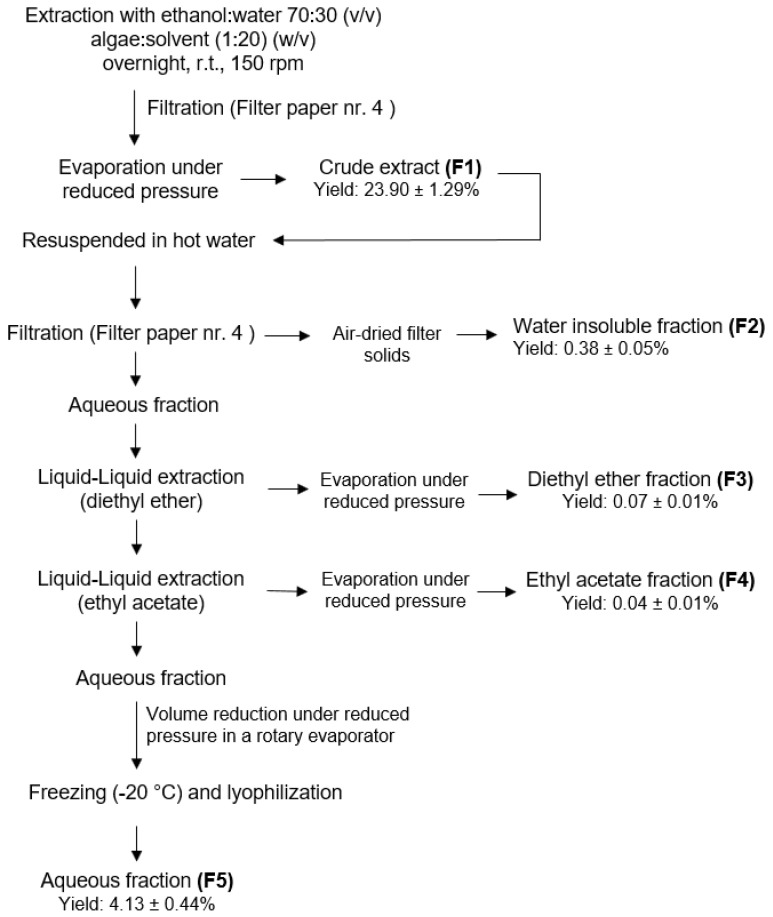
Extraction methodology to obtain *Gelidium corneum* fractions (F1–F5).

To obtain different fractions from *G. corneum*, a sequential extraction methodology was performed (Figure 1). Solvents were selected according to EU Regulation No. 1223/2009 for cosmetic application and were obtained from VWR-BDH Chemicals (Fontenay-sous-Bois, France). Powdered seaweed (100 g) was extracted with ethanol: water (70:30) under constant stirring (150 rpm), over 17 h, protected from light. The hydroethanolic solution was posteriorly filtered (qualitative filter paper (FP) nr. 4, VWR International, Alcabideche, Portugal) and concentrated under vacuum at low temperature (<40 °C) in a rotary evaporator. In total, 20 g of the dried crude extract (F1) was resuspended in Milli-Q water (Advantage A10 Milli-Q lab, Merck, Darmstadt, Germany) previously warmed to 75 °C and filtered (FP nr. 4), affording a solid insoluble fraction (F2) and an aqueous fraction. After cooling to room temperature (r.t.), this last one was subjected to a liquid–liquid partition, firstly with diethyl ether (F3) and then, with ethyl acetate (F4), which were evaporated until dryness in a rotary evaporator. The remaining aqueous fraction (F5) was also evaporated until a half volume was obtained, and then it was frozen (−20 °C) and lyophilized. The extraction procedure flowchart is depicted in Figure 7.

### 4.3. Chemical Screening by ^1^H NMR

A preliminary chemical screening of all fractions (F1–F5) obtained from *Gelidium corneum* was attained by proton nuclear magnetic resonance (^1^H NMR) spectroscopy. Samples (c.a. 5–6 mg) were dissolved in 0.5 mL of deuterated solvents (CDCl_3_, MeOD, or D_2_O) (Sigma-Aldrich, St. Louis, MO, USA) and the ^1^H NMR spectra were recorded at 400.13 MHz on a Bruker AMX400 spectrometer (Bremen, Germany), at 25 °C. Chemical shifts (δ) are expressed in ppm and referenced to the residual solvent signal (δ_H_ 7.26 CDCl_3_, δ_H_ 3.31 MeOD, δ_H_ 4.79 D_2_O).

### 4.4. Antimicrobial Activity of Gelidium corneum Fractions against Microorganisms Associated with Skin Disorders

The antimicrobial activity of *G. corneum* fractions was evaluated against three microorganisms that belong to the skin’s natural microflora, namely, three Gram (+) bacteria, *Staphylococcus epidermidis* (DSM: 1798), *Staphylococcus aureus* (ATCC: 25923) and *Cutibacterium acnes* (DSM: 1897) obtained from the German Collection of Microorganisms and Cell Cultures (DSMZ). The growth conditions and media used for each microorganism were: trypticase soy broth (VWR-BDH Chemicals-Prolabo, Leuven, Belgium) + 0.3 % (*w*/*w*) of yeast extract (VWR-BDH Chemicals-Prolabo, Leuven, Belgium) at 37 °C for *S. epidermidis*; tryptic soy broth (VWR-BDH Chemicals-Prolabo, Leuven, Belgium) in anaerobic conditions at 37 °C for *C. acnes*, and lysogeny broth (LB) medium (VWR-BDH Chemicals-Prolabo, Leuven, Belgium) at 37 °C for *S. aureus*. Fractions were suspended in dimethyl sulfoxide (DMSO) at 100 mg/mL, and the assays were conducted on 96-well microplates with the fractions at a maximum concentration of 1000 μg/mL. Antimicrobial activity was evaluated in the exponential phase of each microorganism. Microorganisms’ growth was accompanied spectrophotometrically at 600 nm, and the results were expressed as a percentage of growth inhibition relative to the control (growth medium with microorganism and vehicle (DMSO)). For the fractions that affected the microorganisms’ growth by more than 50%, a dose–response was conducted (10–1000 μg/mL) and the IC_50_ was determined. Oxytetracycline was used as a positive control.

### 4.5. Mechanisms of Action Underlying the Antimicrobial Activity

To understand the mechanisms that could be underlying the antimicrobial effects, further tests were conducted with the fractions that exhibited the highest activity (inhibition >50% at 1 mg/mL).

#### 4.5.1. Membrane Potential Analysis

The membrane potential variation assay was adapted from Clementi et al. [58], by combining the potentiometric dye bis-(1,3-dibutylbarbituric acid) trimethine oxonol (DiBAC_4_(3)) (Thermo Fisher Scientific, Waltham, MA, USA) with the DNA-staining dye propidium iodide (PI) (Sigma Aldrich, Darmstadt, Germany). The extracts with the highest activity were tested at ½ IC_50_, IC_50_ and 2 × IC_50._

Freshly grown bacteria were centrifuged at 1000× *g* and washed twice with 1× PBS (Sigma-Aldrich, Darmstadt, Germany). The pellet was resuspended in 10 mL PBS buffer at 1.5 MacFarland, and 0.25% (*v*/*v*) of a stock glucose solution (1 M) was added to the mix. A blank was prepared with the same proportions but lacking the microbial solution. The microbial solution was then incubated at 37 °C for 30 min. A solution of carbonyl cyanide 4-(trifluoromethoxy) phenylhydrazone (FCCP) (Sigma-Aldrich, Darmstadt, Germany) was used as the positive control for hyperpolarization. A positive control for total membrane permeabilization was conducted with heat-treated (100 °C, 10 min) bacteria.

After incubation, 45 μL of DiBAC_4_(3) (25 μM) + 90 μL of PI (1 mg/mL) was added to the microbial solution, distributed through 96-well black plates (99 μL per well), and then incubated in the dark at 37 °C, over one hour. The samples were quickly added (1 μL per well) and the fluorescence read at 490/516 nm excitation/emission (DIBAC) and 535/617 nm (PI) in 30 s intervals for 10 min (Multimodal Synergy H1, BioTek^®^ Instruments, Winooski, VT, USA). The results are presented in DiBAC_4_(3) relative fluorescence units (RFU).

#### 4.5.2. Membrane Damage Assay

Membrane damage analysis was performed according to Pinteus et al. [48], as follows: A freshly overnight grown culture was centrifuged (4000 rpm, 5 min) and resuspended in sterile saline solution (0.85%) at an optical density of Abs_600_ = 0.5. Seaweed fractions were added at a concentration of ½ IC_50_, IC_50_ and 2 × IC_50_, and incubated over 4 h at 37 °C for *S. epidermidis*, and 6 h for *C. acnes*. DMSO was used as a negative control. Blanks were prepared with samples without microorganism. A positive control was prepared with a thermic treatment (100 °C, 10 min) to induce total membrane permeability. All suspensions were transferred to a black microplate and incubated with 2 μM Sytox Green (Thermo Fisher Scientific, Waltham, MA, USA) for 10 min in the microplate reader. The resultant fluorescence of the DNA-bound dye was quantified on a fluorescence microplate reader (Multimodal Synergy H1, BioTek^®^ Instruments, Winooski, VT, USA). The membrane damage was determined in % of control (DMSO).

#### 4.5.3. DNA Damaging Potential

DNA damaging potential was assessed following the methodology described by Hu et al. [59]. Plasmid (pGADT7—7987 bp) DNA (5 µL; 100 ng) was mixed with seaweed fractions (2 µL; 10 mg/mL) and ultrapure water (13 μL). The reaction mixture was incubated at 37 °C for 1 h before being loaded onto a 0.8% agarose gel containing 1% RedSafe^TM^. Gene Ruler 1 Kb DNA Ladder (5 µL) was also loaded onto the gel. Electrophoresis was then performed for 60 min under 85 V. DMSO was used in the same conditions as a negative control and ciprofloxacin (10 and 30 µg/mL) was used as positive control.

### 4.6. Data and Statistical Analysis

To determine possible significant differences relative to the control, a one-way analysis of variance (ANOVA) with Dunnett’s multiple comparison test was used. All data were checked for normality (Shapiro–Wilk test) and homoscedasticity (Levene´s test). When requirements for an ANOVA were not met, a non-parametric Kruskal–Wallis and Dunn’s multiple comparison test were applied. The IC_50_ values were determined using the GraphPad v9.3.1 software through the equation y = 100/(1 + 10 ^(X−Log IC^_50_^)^). Calculations were carried out and final graphical representations were made using GraphPad v9.3.1 (GraphPad Software, La Jolla, CA, USA). All data were obtained from at least three independent experiments carried out in triplicate and are presented as standard error of the mean (SEM), with a significance level of 0.05 (*p* < 0.05).

## 5. Conclusions

With the increasing awareness on the importance of sustainable strategies for the development of new nature-based health and wellness products, this work addressed, for the first time, the potential of *Gelidium corneum* as a source of antimicrobial compounds for skin dysbiosis-related diseases. This seaweed is particularly interesting since it is already explored industrially for agar extraction. Thus, within a circular economy concept, it would be of high economic relevance to define a biorefinery concept to extract both antimicrobial compounds and agar, thus taking the maximum advantage of the same natural resource for maximum environmental and economic sustainability.

The results reveal that *G. corneum* contains compounds with high antimicrobial activity against two important skin opportunistic pathogens, *C. acnes* and *S. epidermidis.* The compounds concentrated in F2 (water insoluble fraction) and F3 (diethyl ether fraction) seem to affect both microorganisms’ growth by inducing changes in membrane polarization and by binding to DNA, promoting damage. This dual mechanism of action may provide therapeutic advantages for the treatment of skin dysbiosis-related diseases.

## Figures and Tables

**Table 1:** Dose–response analysis of *Gelidium corneum* F2 (water insoluble) and F3 (diethyl ether) fractions against *Staphylococcus epidermidis* and *Cutibacterium acnes*.

Fraction	*Staphylococcus epidermidis*	*Cutibacterium acnes*
	IC_50_ (µg/mL)	
F2	53.29 (48.75–57.91)	16.10 (7.27–23.02)
F3	102.80 (87.15–122.30)	51.04 (43.36–59.74)
Oxytetracycline	12.40 (11.22–16.13)	0.07 (0.05–0.09)

## Data Availability

Not applicable.
